# *De novo* genome assemblies of threatened Asian hornbills (Bucerotidae) reveal declining population trajectories during the late Pleistocene

**DOI:** 10.1186/s12862-026-02547-3

**Published:** 2026-06-27

**Authors:** Pooja Yashwant Pawar, Gopi Krishnan, Rohit Naniwadekar, Jahnavi Joshi

**Affiliations:** 1 Manipal Academy of Higher Education, Manipal, India; 2 Nature Conservation Foundation, Mysore, India; 3 Centre for Cellular and Molecular Biology, Hyderabad, India; 4 Academy of Scientific and Innovative Research, Ghaziabad, India

**Keywords:** Conservation genomics, Asian hornbills, Demographic history, Hybrid assembly

## Abstract

**Background:**

Asian hornbills are flagship species of the wet tropics that face significant threats from hunting, habitat loss, and fragmentation. Despite being conservation flagships, whole genome information is available for only two of the 32 Asian hornbill species. In this study, we provide the first de novo genome assemblies for four hornbill species (Bucerotidae) in Asia.

**Methods:**

We used a combination of long-read and short-read sequencing data to assemble and annotate de novo hybrid genomes of four species of hornbills. We also assembled and compared mitochondrial genomes of these species. Using a comparative genomics approach, we performed orthology assignment and gene evolution analyses to identify unique gene families in Asian hornbills, gene families that showed significant expansion, their functions and structural variation. Furthermore, using the Pairwise Sequentially Markov Coalescent (PSMC) method, we reconstructed demographic histories of hornbill species to examine changes in their population trajectories in the past.

**Results:**

We present hybrid genome assemblies for Great Hornbill (B. bicornis - GH), Rufous-necked Hornbill (A. nipalensis- RNH), Malabar Pied Hornbill (A. coronatus- MPH) and Wreathed Hornbill (R. undulatus- WH). The genome sizes of these hornbills range from 1.1 Gb to 1.3 Gb, with over 95.9% completeness and gene prediction BUSCO. We reported 10,525 orthogroups shared among four Asian hornbill species and identified significant expansion in gene families associated with structural keratin development in Asian hornbills compared to their ancestors. We also provide annotated mitogenomes for each of these species. Furthermore, we found that the WH, a more abundant, widely distributed, and migratory species, showed a higher Ne than the other three hornbill species. However, an overall decline in Ne for all species was recorded during the Pleistocene climatic fluctuations.

**Conclusions:**

We present the first-ever, high-quality reference genomes for the threatened hornbill species from Asia. Hornbills have shown significant expansion in genes involved in structural keratin development. Our results indicate that Pleistocene climatic fluctuations have led to dramatic population declines in all four species. We believe that this study provides robust genomic resources to support future comparative and conservation genomics efforts for hornbills.

**Supplementary Information:**

The online version contains supplementary material available at 10.1186/s12862-026-02547-3.

## Introduction

Hornbills (Order: Bucerotiformes) are avian flagships of tropical Asia and Africa, occupying a wide range of habitats ranging from African savannas to the wet evergreen forests of Southeast Asia [1]. While savanna-dwelling hornbills are primarily carnivorous, forest-dwelling species are predominantly frugivorous, playing a crucial role in seed dispersal and the regeneration of tropical forests [2]. Unfortunately, hornbills face severe threats from hunting and forest loss [3, 4], resulting in isolated populations that often occur in low densities [5–7]. Consequently, many Asian hornbill species, especially island endemics, are likely to have low genetic diversity due to small populations and isolation. For example, a recent mtDNA study on the endemic Narcondam Hornbill (*Rhyticeros narcondami*) revealed extremely low nucleotide diversity, highlighting the value of genomic tools in understanding hornbill populations [8]. While molecular phylogenetic studies have clarified evolutionary relationships among hornbills, some remain unresolved and could benefit from genomic work [9]. Genomic data could also provide information on molecular underpinning for trait variation in hornbills, which differ in morphology, colour, and vocalisations.

Partial mitogenome characterizations have been helpful for understanding genetic diversity and gene flow in endangered hornbills [10]. For example, mitogenomes revealed no genetic differentiation between captive Great Hornbills (*Buceros bicornis*) across five zoos in Thailand, highlighting their utility in ex-situ conservation [11]. Thus, genomic information can be critical for the management and conservation prioritisation of these rare species.

Interestingly, genomic data also allows for inferring the demographic histories of a species, which can provide valuable insights into how past climatic changes or anthropogenic disturbances might have influenced population dynamics. Rhinoceros Hornbill *Buceros rhinoceros* exhibited declines in effective population size during the Pleistocene [12]. Given that hornbills show strong habitat preferences and have been targeted by hunting for at least the last 50,000 years, they make an ideal candidate group for exploring population demography over historical time scales.

Of the 62 extant hornbill species, the genomes of two Asian hornbill species (Rhinoceros Hornbill *B. rhinoceros*; Accession no. ASM71030v1 and Great Hornbill; ASM2756380v1), and 15 species from African hornbill species (ASM4729193v1, ASM4729177v1, ASM4676605v1, ASM4729227v1, ASM4729169v1, ASM4729180v1, ASM4729225v1, ASM4729195v1, ASM4729166v1, ASM4676607v1, ASM4676609v1, ASM4729219v1, ASM4729197v1, ASM4774805v1) have draft genomes assembled using only short-read sequencing data, except for two species of ground hornbill. Additionally, the mitochondrial genomes of the Writhed-billed *Rhabdotorrhinus waldeni*, Visayan *Penelopides panini*, and Great and Wreathed Hornbills have been sequenced [13–15]. The lack of good quality reference genomes for most of the hornbill species is a limiting factor in further exploring genomics and evolutionary ecology of such ecologically significant yet threatened birds.

Like other Asian Hornbills, Great Hornbill *Buceros bicornis* (hereafter GH), Wreathed Hornbill *Rhyticeros undulatus* (hereafter WH), Malabar Pied Hornbill *Anthracoceros coronatus* (hereafter MPH), and Rufous-necked Hornbill *Aceros nipalensis* (hereafter RNH) face alarming threats from rapid habitat loss, habitat destruction, hunting and illegal wildlife trade across their distribution ranges [2–4]. The GH, WH, and RNH are categorised as ‘Vulnerable’ in the IUCN Red List and are included in the CITES, while the MPH is classified as ‘Near Threatened’. Conserving these birds remains a challenge across their range-distribution countries. While the GH, WH, and RNH prefer evergreen forests, the MPH prefers the moist deciduous forests of the Indian subcontinent. Given their differences in habitat preferences, they offer contrasts for investigating population demographics in the historical context, especially since South and Southeast Asia have experienced significant fluctuations in the extent of wet and dry forests in the Pleistocene [16, 17].

**Fig. 1:**
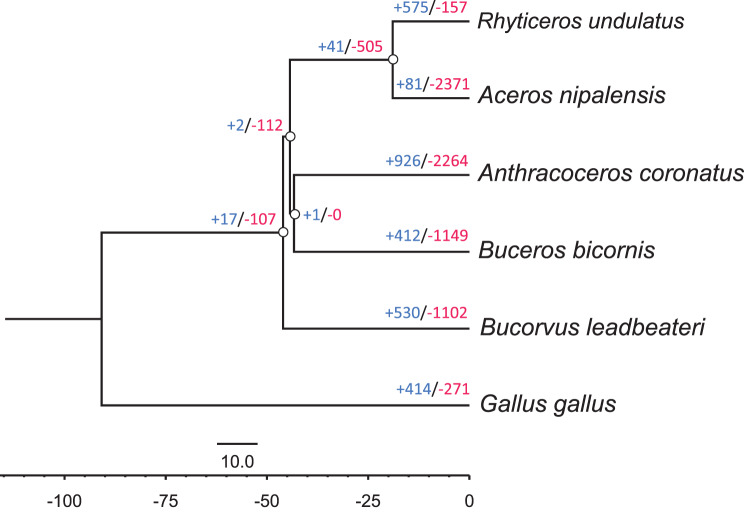
Ultrametric phylogenetic tree depicting evolutionary relationships and gene family expansions (numbers in blue colour) and contractions (numbers in red colour) among select Asian hornbill species

In this study, we present high-quality assembled and annotated genomes of four threatened Asian hornbill species using hybrid approach: GH, WH, MPH, and RNH (Fig. 1), representing distinct ecological and biogeographic contexts. Using one high-quality genome of Southern Ground Hornbill *Bucorvus leadbeateri* (hereafter SGH) [18] and genomes from this study, we identified unique gene families and their evolution in Asian and African hornbills. In addition to nuclear genomes, we annotated and compared their mitochondrial genomes. Finally, we reconstructed demographic histories using whole-genome data to investigate how historical climatic fluctuations may have shaped population trajectories across species differing in habitat preferences and distributions. This study provides foundational genomic resources for hornbills and offers insights into the evolutionary and demographic history of threatened and ecologically important species.

## Materials and methods

### Sample collection and DNA extraction

We collected tissue samples of GH (*n* = 2), WH (*n* = 2), RNH (*n* = 3), and MPH (*n* = 1) from wild and captive individuals. The GH sample was obtained from a deceased captive individual at Nehru Zoological Park, Hyderabad. One GH sample was from the Western Ghats in north Karnataka, collected in the wild. WH samples were collected from trophies in eastern and western Arunachal Pradesh, India and three RNH samples were collected from trophies in eastern, central and western Arunachal Pradesh in the Eastern Himalayan region. The MPH sample was collected from a deceased wild individual from the Western Ghats region of Maharashtra. Sample collection was carried out with prior approval from the Research Ethics Committee (NCF-EC-01/12/2022-(73)) and valid research permits from the state forest departments (CWL/GEN/2018–19/Pt.IX/NG/365–68; MSBB/Desk-5/Research/652/2023–24; Desk −22(8)/WL/CR-68(19–20)/397723-24, PCCF(WL)/E2lCR-1 1, 12,022–23). Each sample was stored in absolute ethanol and stored at −20 °C in the laboratory. We carried out high molecular weight DNA extraction using the Phenol-Chloroform-Isoamyl alcohol method (see supplementary section 1) for Oxford Nanopore sequencing and the QIAGEN DNeasy Blood & Tissue kit for Illumina sequencing.

### Library preparation, sequencing, and read quality check

Library preparation for both Illumina and Oxford Nanopore platforms and sequencing was done at the Next Generation Sequencing (NGS) Facility at the CSIR-Centre for Cellular and Molecular Biology, Hyderabad, India. Libraries for long-read sequencing were prepared using Ligation Sequencing Kit V14 (SQK-LSK114, ONT) by following the manufacturer’s protocol and sequenced individually on a Oxford Nanopore PromethION sequencer using PromethION Flow Cell R10.4.1. Each sample was processed separately on the sequencer. For short-read sequencing, libraries were prepared using TruSeq DNA PCR-Free kit (Illumina) by following the manufacturer’s protocol. The short-read (150 bp paired-end) sequences were generated by multiplexing samples on a S4 flow cell on the Illumina NovaSeq 6000 sequencer. We examined the sequencing quality for long-read and short-read data. The Phred quality score threshold was set at nine for long-read data. For the paired-end short-read data, we filtered out duplicates and low quality reads (sequences < 50 bp and reads containing ambiguous bases) using an accuracy threshold of Q20 with Fastp (v 0.23.4) [19].

### Genome assembly

GH, WH, RNH, and MPH de novo reference genomes were assembled using a hybrid approach that combined long-read (ONT) and short-read (Illumina) sequencing data. We used the single-individual genome assembly approach for each species. We assembled the genome of GH using Canu v. 2.3 [20], and WH, MPH, and RNH using HifiAsm v. 0.25.0 [21] with long-read sequence data. All four long-read genome assemblies obtained from ONT long-read data were polished using Illumina short-read data with POLCA (from Masurca v. 4.1.0) [22]. The POLCA (POLishing by Calling Alternatives) allows the use of high-accuracy short-read data to identify and correct base substitution mismatches and small indels that remain in the draft genome assembly generated using long-read data. This hybrid approach helps in improving the overall accuracy of the assembly. We evaluated genome assembly completeness using Benchmarking Universal Single-Copy Orthologs (BUSCO v 6.0.0) [23] with the Aves ortholog database (aves_odb12), which contains 6251 genes. The assembly statistics were generated using BlobToolKit v 4.4.5 [24], and the output was visualized as snail plots. We also evaluated genome assembly quality using k-mer based quality metrics using Merqury v 1.3 [25].

### Repeat masking and genome annotation

To mask the repeat content of the genome, we performed *de novo* repeat prediction using RepeatModeler v. 2.0.5 [26]. We built custom libraries for four genomes using the curated Dfam database and LTRStruct pipeline [26, 27]. We then soft-masked the polished genomes with these custom libraries using RepeatMasker v. 4.1.5 [26].

Gene annotation was performed using GALBA v. 1.0.11 [28], which uses miniprot in combination with AUGUSTUS. For this, we created a reference protein dataset of 7,197,644 protein sequences of Aves from the Uniprot and Orthodb databases, which was used in the GALBA pipeline to predict protein-coding sequences in the soft-masked genome. Next, we removed unsupported predictions that are likely algorithmic artefacts lacking alignment with the protein databases. We further filtered out multiple isoforms, retaining only the longest isoform for each gene, forming the final annotated gene dataset.

We evaluated the completeness of the gene annotation using BUSCO Orthologs (v 6.0.0) with the Aves ortholog database (aves_odb12), which contains 6251 genes [23]. We further assessed the completeness and consistency of the gene annotation using OMArk (Orthology Mapping Annotation) v. 2.0.3 with the whole OMA database (LUCA.h5) as reference [29]. Similar to BUSCO, OMArk evaluates the completeness of gene prediction by calculating the proportion of expected ancestral genes from a taxonomic group that are present in the query annotated genome. Additionally, OMArk assesses the taxonomic consistency of predicted genes based on homology relationships. Genes lacking homology to known orthologous groups are classified as “unknown” or “noise”.

### Orthology assignment and gene family evolution analysis

Using OrthoFinder v. 2.5.5, we performed orthology assignment on gene prediction results from GALBA of Asian hornbills (GH, WH, MPH and RNH) and African (SGH) hornbill and *Gallus gallus* [30]. This analysis identified the number of orthogroups per species. The assigned orthogroups were visualised as a Venn diagram showing the numbers of unique and shared orthologous Asian hornbills using Orthovenn3 [31].

We used CAFE5 v. 4.2.1 to examine multicopy gene families that underwent significant expansion or contraction [32]. For CAFE analysis, we provided a dated phylogenetic tree from TimeTree5 [33] with *Gallus gallus* as the outgroup for all hornbill species, along with a gene count file from the OrthoFinder.

The number of significantly evolved genes from CAFE analysis was visualised on a phylogenetic tree using CafePlotter v. 0.2.0 (https://github.com/moshi4/CafePlotter). The significantly evolved gene families which differed from neutral patterns were analysed separately for functional annotation using EggNOG-mapper v. 2.1.14 [34].

### Single nucleotide polymorphisms and structural variants

We compared the published GH genome from NCBI (ASM2756380v1) with the four genomes assembled in this study to calculate structural variants (SVs). Using the default settings in MUMmer v 3.23 [35], we estimated SNPs and SVs by aligning each assembled genome with ASM2756380v1 with Assemblytics v1.2.1 [36]. For single nucleotide polymorphism (SNP) identification, trimmed short-reads were aligned to the reference genome using default settings in BWA-MEM. The resulting alignments were converted to BAM format and sorted using Samtools v1.9.67. PCR and optical duplicates were identified and marked using Picardtools MarkDuplicates (http://broadinstitute.github.io/picard). SNP calling was performed using mpileup in BCFTools v1.21 [37], and variant filtering was carried out using VCFtools v3.0 [38]. Indels were excluded, and only SNP loci with a minimum Phred-scaled base quality of 30, a minimum genotype quality of 30, and a minimum minor allele count of 3 were retained. We identified SNPs for two GH, three WH and three RNH individuals by mapping short-read data to the reference genomes assembled in this study. We could not reliably estimate SNPs for MPH since MPH genome data were available only for a single individual.

### Mitogenome assembly

We assembled and analyzed the mitogenome from short-read data with the packages GetOrganelle [39], MITOS2 [40] web server and MitoZ v. 3.6 [41] to assemble, annotate and visualize mitogenomes of the four hornbill species. GetOrganelle was used to extract mitochondrial sequences from the reads, MITOS2 was used to annotate the sequences obtained from the GetOrganelle result and mitogenomes were visualised using MitoZ. We assembled mitogenomes for all four species in this study. In addition, published mitogenomes were available for GH [14] and WH [15], which were included for comparative analyses to assess variation between assemblies generated in this study and previously published assemblies. We estimated and compared GC content and GC skewness (G - C)/(G + C) across the total genome length, protein-coding genes, rRNA, and tRNAs within and across species [42].

### Demographic history

We reconstructed demographic histories for all four species of hornbill using the Pairwise Sequentially Markovian coalescent (PSMC) method [43]. We removed scaffolds that mapped to the sex chromosomes from the genome assembly by comparing/referencing with the *Gallus gallus* genome (Accession number: GCA_016699495.1). Illumina short-reads for 2 GH, 3 WH, 4 RNH and 1 MPH individuals were mapped to the autosome-only assembly of respective hornbill species. The mapping was done using default settings in BWA-MEM. The resultant alignments were used for the variant calling and filtering SNPs (as detailed in Section “Single nucleotide polymorphisms and structural variants”). We assessed the quality of mapping by estimating average genome coverage using QualiMap v 2.2.2 [44]. The parameters for PSMC analysis were -N25 -t5 -r5 -p ‘25 × 2+4+4+6, where N is the number of iterations, t is the upper limit of time to the most recent common ancestor, r is the ratio of the scaled mutation rate and the recombination rate, and p is the number of free atomic time intervals. The default parameter values in the PSMC model are for humans [43], hence, our model is based on published PSMC studies on birds [45, 46]. We bootstrapped the PSMC run 100 times to estimate the variance in effective population sizes. PSMC analysis was performed using the Pairwise Sequentially Markovian Coalescent (PSMC) software [43], available at the GitHub repository (https://github.com/lh3/psmc). Generation time used for GH, WH, MPH, and RNH was 14.1, 8.6, 5.3, and 9.5 years [47], respectively; and the mutation rate was 7 × 10^−9^ mutations per generation [12]. It is important to note that PSMC analysis provides the estimated effective population size which maintains a very small variance and high accuracy, specifically between 20 kya and 3 mya. Beyond these limits, the less number of recombination events, lead to poor accuracy [43]. We performed PSMC analysis on multiple individuals of GH (*n* = 2), WH (*n* = 3) and RNH (*n* = 3). One GH individual from the Western Ghats and another from the Eastern Himalaya were sampled. Two WH individuals from eastern and western Arunachal Pradesh each and three RNH individuals were sampled from eastern, western and central Arunachal Pradesh in the Eastern Himalayan region. The MPH was sampled from the Western Ghats.

## Results

### Genome sequencing and whole genome assembly

For GH, we obtained 26 GB (26x coverage) of long-read data comprising 4 million reads. The long-read data obtained for WH was 51 GB that comprised 9.5 million reads (54x coverage), for RNH 30 GB data with 12.7 million reads (30x coverage), and for MPH (GB data yielded 1.1 million reads (9x coverage). The GC content of the long-read data ranged between 44 and 45% across all four species. The mean read lengths ranged between 2991 bp to 5729 bp. The Phred quality scores ranged between 12.54 to 22.67. For GH, a total of 436 million paired-end short-reads were generated. After removing 9.3% duplications, we retained 429 million reads, resulting in 63x coverage of the short-read data. The mean read length was 148 bp, and the GC content was 45.49%, with 96.3% of bases having a Phred quality score greater than or equal to Q20. Similarly, for WH, MPH, and RNH, we obtained 65x, 119x, and 66.6x coverage of short-read data, respectively. The mean read length was 148 bp, and the number of reads generated was 442 million for WH, 882 million for MPH, and 493 million for RNH. The GC content across all three species was 45%.

**Fig. 2:**
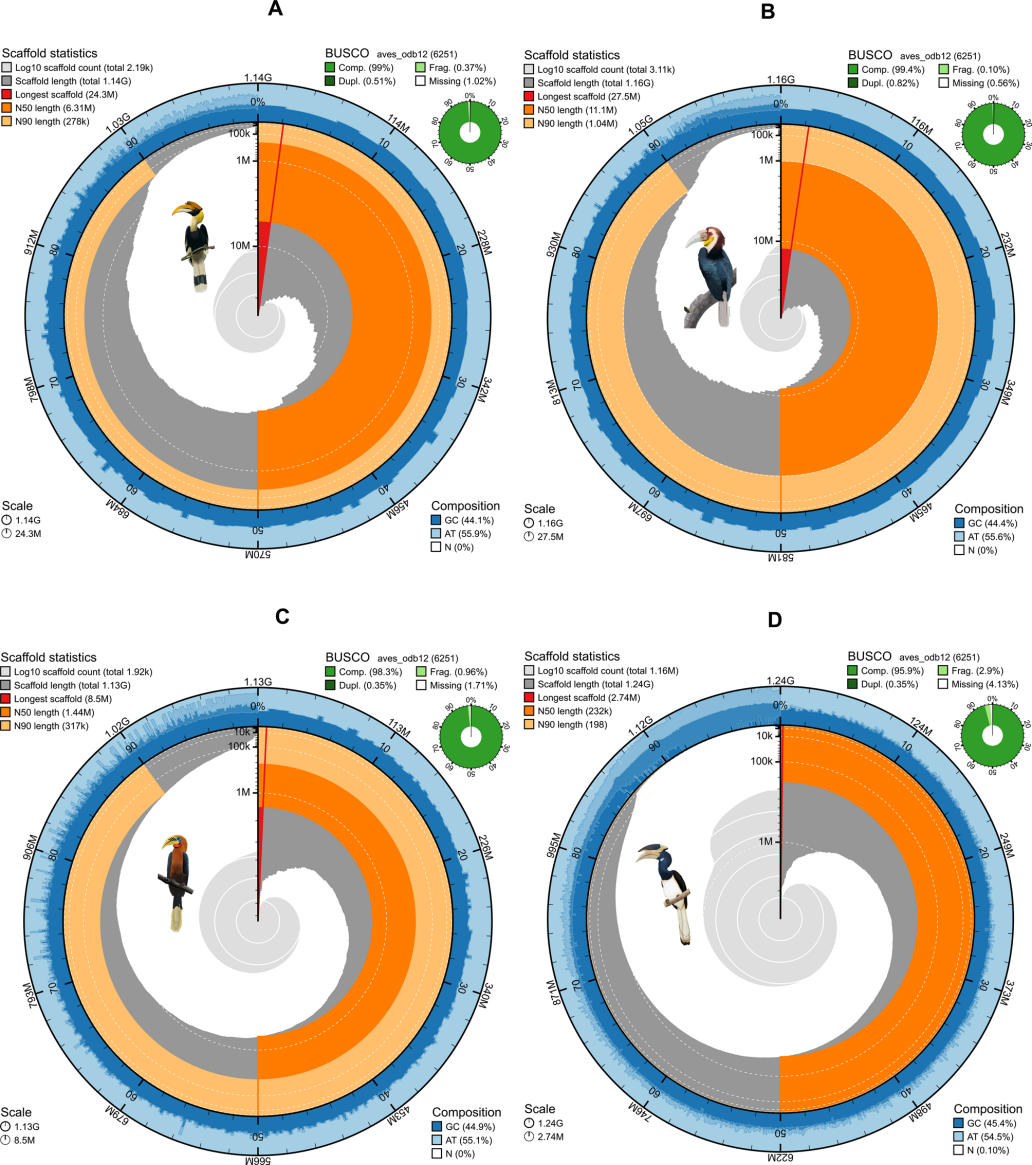
Genome assembly snailplots showing assembly stats sequence composition and BUSCO completeness score for **A**) Great hornbill, **B**) Wreathed hornbill, **C**) Rufous-necked hornbill, and **D**) Malabar Pied hornbill, respectively. (hornbill illustrations by Bhagyashri Patwardhan)

The genome size of the *de novo* assembled GH, WH, MPH, and RNH genomes were 1.14 Gb, 1.16 Gb, 1.24 Gb, and 1.13 Gb, respectively (Fig. 2). The total number of assembled contigs was 2,192 for GH, 3,114 for WH, 23,027 for MPH and 1922 for RNH; with contig N50 values being 6.3 Mb for GH, 11.3 Mb for WH, 0.23 Mb for MPH and 1.4 Mb for RNH, suggesting that three assemblies are highly contiguous. BUSCO evaluation to assess the genome completeness predicted the polished assembly of GH, WH, MPH and RNH to be 99%, 99.4%, 95.9% and 98.3% complete The detailed assembly statistics are given in Table 1. The k-mer based quality metric showed 99.9% correctness with QV values 39.8, 41.9, 34.1 and 69.2 for GH, WH, RNH and MPH assemblies respectively.

**Table 1:** Genome assembly statistics for four species of hornbills

	Great Hornbill (GH)	Wreathed Hornbill (WH)	Rufous-necked Hornbill (RNH)	Malabar Pied Hornbill (MPH)
*Genome assembly statistic*				
No. of long-reads	4 M	9.5 M	12.7 M	1.1 M
Coverage* (long-reads)	26 x	54 x	30 x	9 x
Mean read length (bp)	4713	4916	2991	5729
Mean read quality score	12.54	18	20.15	22.67
No. of short-reads	429 M	442 M	493 M	882 M
Coverage* (short reads)	63 x	65 x	66.6 x	199 x
Genome size	1.14 Gb	1.16 Gb	1.13 Gb	1.3 Gb
Number of contigs	2192	3114	1922	23027
N50	6.3 Mb	11.3 Mb	1.4 Mb	231.9 Kb
Largest contig length	24.3 Mb	27.5 Mb	8.5 Mb	2.7 Mb
GC content	44.08%	44.41%	44.85%	45.4%
% single copy genes identified using BUSCO	98.5%	98.6%	97.9%	95.5%
BUSCO completeness	99.0%	99.1%	98.1%	95.9%
*Repeat masking statistics (%)*				
Retroelements -SINEs -LINEs	6.05 0.04 4.03	7.83 0.05 3.83	4.94 013 3.52	4.92 0.07 3.63
DNA transposons	0.10	0.07	0.16	0.10
Unclassified	2.89	2.03	2.35	10.79
Small RNA	0.37	0.15	0.1	0.05
Satellites	0.03	0.04	0.01	0.05
**Total bases masked**	**9.54**	**10.13**	**7.48**	**15.88**

The coverage is calculated as the total number of bases sequenced divided by the genome length

### Genome annotation and gene family evolution

A total of 9.54%, 10.13%, 15.88% and 7.48% of genome sequences were annotated and masked as repeat sequences for GH, WH, MPH, and RNH, respectively. The repeat regions were predominantly composed of Retroelements, accounting for 6.05%, 7.83%, 4.92%, and 4.94% of the genomes in GH, WH, MPH, and RNH, respectively. The composition of repeat content and the detailed repeat statistics are given in Table 1.

GALBA predicted 21,316, 21,357, 22,222 and 14,549 protein-coding genes for GH, WH, MPH and RNH, respectively. BUSCO analysis to assess the completeness of protein predictions showed that the annotations were 93.3% complete for GH, 95.9% for WH, 68.1% for MPH and 86.7% for RNH. Similarly, the OMArk-based completeness assessment using 11,063 conserved Hierarchical Orthologous Groups estimated annotation completeness to be 94.63% for GH 94.6% for WH, 90.35% for MPH and 88.24% for RNH. See Supplementary Table 6 for detailed OMArk statistics.

**Fig. 3:**
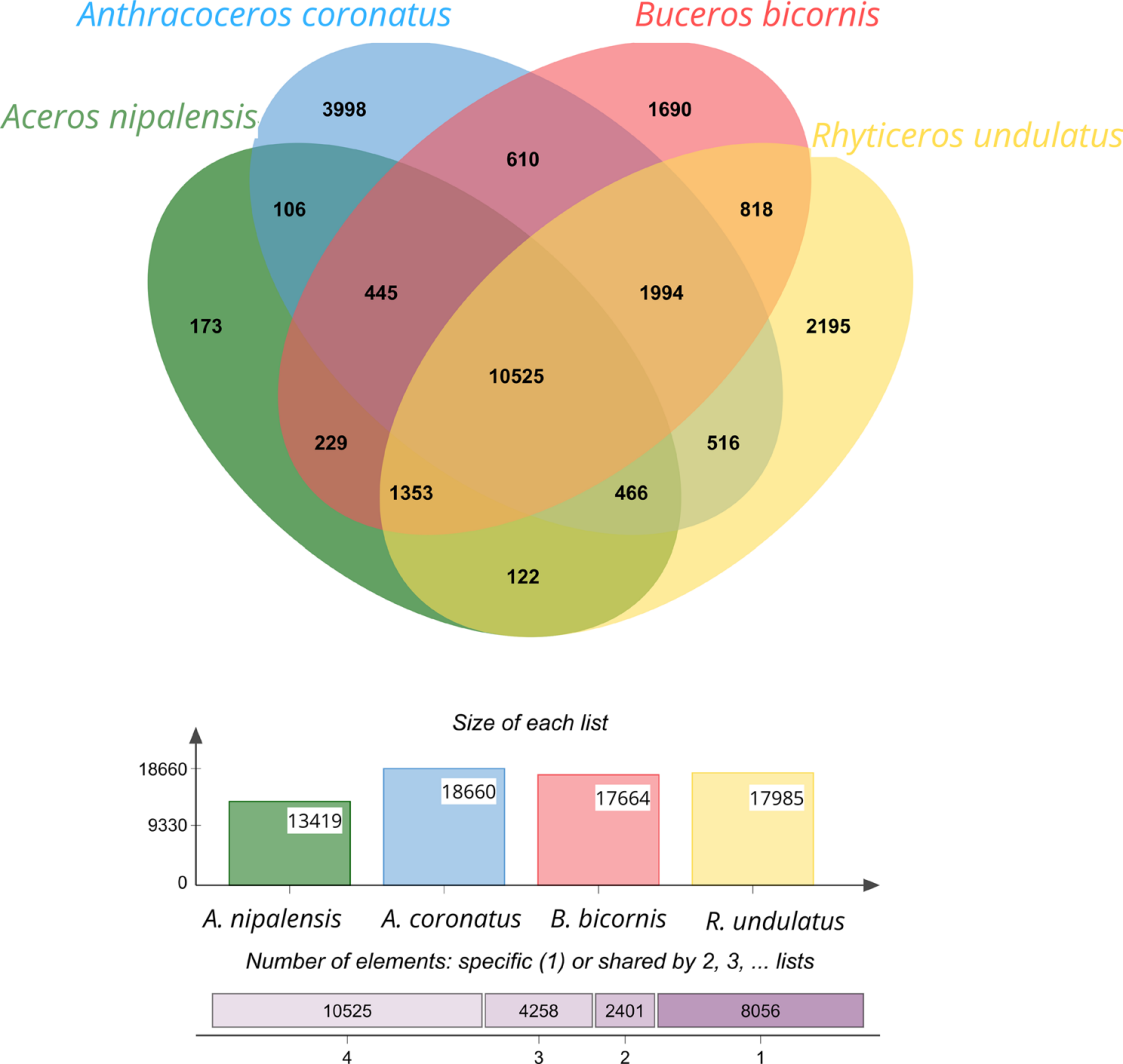
Venn diagram showing unique and shared orthologous gene families in Asian hornbills. The accompanying bar graphs summarize the number of orthologous gene families identified for each species

Across six genomes (four Asian hornbills from this study, one African hornbill [18], and Chicken (*Gallus gallus*), 1,13,132 genes were identified, of which 91.1% were assigned to orthogroups. Overall, 18,882 orthogroups were identified (mean orthogroup size = 6). Among Asian hornbills, 10,525 orthogroups were shared across species, including 8,056 single-copy orthologs (Fig. 3). Gene family evolution analysis showed that, out of 14,806 gene families analysed, 997 gene families evolved (expanded or contracted) significantly relative to their ancestral states (Fig. 1). Asian hornbills showed significant expansion in two gene families compared to their ancestral African hornbill lineage. These expanded families were associated with: a) development of structural keratin (feathers, beak, claws, etc), including genes related to feathers, beak, and claw morphology, b) reproductive functions, including fertilisation-related processes. These gene families showed 5-fold and 7-fold increased gene count, respectively.

### Single nucleotide variation and structural variants

We identified single nucleotide variations (SNPs) and structural variants (SVs) across all the hornbill genomes by comparing them against the published *B. bicornis* reference genome (ASM2756380v1) (Supplementary Table 1). In GH, we identified over 1.9 million SNPs and 22,896 SVs comprising 15.6 Mbp of the genome. The predominant structural variation was repeat contractions (50–500 bp), with a total count of 4,938. By comparing with the published *B. bicornis* (ASM2756380v1) genome, for WH, we identified over 53 million SNPs and 49,983 SVs, amounting to 38.4 Mbp, for RNH, we identified 69 million SNPs and 54,455 SV amounting to 38.88 Mbp, and for MPH we identified 63 million SNPs and 30,778 SVs amounting to 5.5 Mbp. In the genomes of these four species, deletions (50–500 bp) were the most common type of structural variation, with total counts ranging between 11,213 to 15,195. On mapping individual data to the species-specific reference genomes, we obtained 2.9 million SNPs for RNH (*n* = 3), 2. million SNPs for GH (*n* = 2) and 10 million SNPs for WH individuals (*n* = 3).

### Mitogenome assembly

The mitochondrial genome assemblies for GH, WH, MPH, and RNH were 15,552 bp, 15,794 bp, 15,569 bp, and 15,622 bp in size, respectively. All four mitogenomes comprised 13 protein-coding genes, 2 rRNAs, and 22 tRNAs (Supplementary Fig. 1). For the detailed mitogenome organization see supplementary Table 2–6. The overall GC and A + T content for the GH mitogenome were 46.9% and 53.1%, respectively. For WH, the GC and A+T content was 48.7% and 51.3%, respectively, for MPH, 47.7% and 52.3%, and for RNH, 48.6% of GC and 51.4%, respectively. The GC content was similar to the concatenated composition of the protein-coding genes, tRNA, and rRNA for the respective hornbill species. The mitogenomic structures were highly conserved between and across species as we did not see differences in GC contents of protein-coding genes, rRNA, tRNA and their respective GC skewness (Table 2.).

**Table 2:** Intra- and inter-specific comparison of mitogenomic structures in four hornbill species

	Great Hornbill (GH)		Wreathed Hornbill (WH)		Rufous-necked Hornbill (RNH)	Malabar Pied Hornbill (MPH)
Accession number	NC_038201	this study	NC_039950	this study	this study	this study
**Total size (bp)**	**17117**	**15552**	**17842**	**15794**	**15622**	**15569**
GC%	46.2	47.4	47.3	48.7	48.6	47.7
AT%	53.7	52.6	52.6	51.3	51.4	52.3
GC skew	−0.392	−0.40	−0.379	−0.39	0.40	−0.40
**PCGs size (bp)**	**11398**	**11402**	**11395**	**11400**	**11405**	**11401**
GC%	47.03	47.2	49.37	49.32	48.93	48.16
AT%	52.97	52.8	50.63	50.68	51.07	51.84
GC skew	−0.424	−0.421	−0.414	−0.414	−0.420	−0.401
**rRNA size (bp)**	**2523**	**2566**	**2534**	**2573**	**2580**	**2568**
GC%	47.96	47.9	49.05	49.1	48.8	47.5
AT%	52.04	52.1	50.95	50.9	51.2	52.5
GC skew	−0.16	−0.16	−0.184	−0.18	−0.20	−0.17
**tRNA size (bp)**	**1556**	**1555**	**1563**	**1565**	**1560**	**1563**
GC%	43.06	42.8	44.69	44.60	44.62	42.99
AT%	56.94	57.2	55.31	55.40	55.38	57.01
GC skew	0.012	0.006	−0.007	−0.008	−0.011	0.0

### Demographic history

**Fig. 4:**
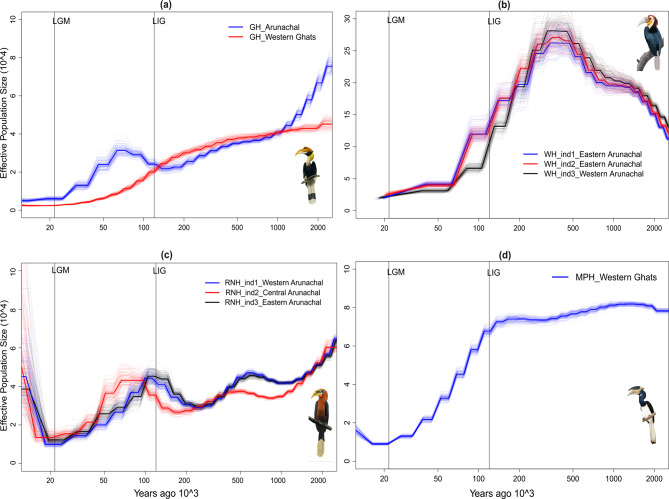
Demographic trends in effective population sizes (Ne) for four hornbill species. The box indicates the Pleistocene epoch (2.5 mya to 11.7 kya), and the vertical lines indicate the beginning of the last interglacial (LIG) and last glacial maximum (LGM). **a**) Great hornbill *Buceros bicornis*; **b**) Wreathed hornbill *Rhyticeros undulatus*; **c**) Rufous-necked hornbill *Aceros nipalensis*; and **d**) Malabar Pied hornbill *Anthracoceros coronatus.* (hornbill illustrations by Bhagyashri Patwardhan)

We estimated the demographic histories of GH, WH, MPH, and RNH using genome assemblies. We observed notable differences in the demographic histories of four hornbill species (Fig. 4).

For GH, the effective population size (Ne) estimates around 15 kya were 12,782 individuals in the Himalayan population and 7,755 individuals in the Western Ghats population. The GH populations in the Himalaya and the Western Ghats reached their peak Ne at the beginning of the Pleistocene (2.5 mya), with 89,138 and 45,157 individuals, respectively (Fig. 4a). For WH, Ne estimates in eastern Arunachal Pradesh ranged between 31,936 and 36,252 individuals at around 20 kya, while the western Arunachal population had a Ne of 36,049 individuals. The effective population sizes for WH peaked at ~0.4 mya, with populations ranging from 2,62,083 to 2,81,166 individuals (Fig. 4b). It was followed by sharp declines for all three individuals throughout the last glacial period (120 kya to 11.9 kya). The RNH populations exhibited multiple fluctuations in the Ne over time, with peak Ne values between 69,893 to 73,219 individuals occurring approximately 2.5 million years ago (Fig. 4c). The MPH population from the Western Ghats had a Ne of 22,591 individuals at around 15 kya. The MPH population exhibited a relatively stable Ne trajectory until the Last Interglacial (LIG, ~120 kya), peaking with 81,833 individuals.

## Discussion

The high-quality, annotated genome assemblies of the four hornbill species provided here will be valuable for future evolutionary and population genomics research on hornbills throughout Asia and Africa. The genome assemblies are robust with moderate to high sequencing coverages and exhibit high completeness. These are among the first hybrid genome assemblies of GH and WH. The contig N50 values for GH and WH exceed the average scaffold N50 reported for published bird genomes [48]. The total genome lengths for all four species fell well within the expected genome size (~1–2.1 Gb) for birds [48, 49]. The genome lengths of Asian hornbills presented in this study are comparable to the genomes of hornbill species (1.1 Gb) in Africa (NCBI: ASM4729193v1, ASM4729177v1, ASM4676605v1, ASM4729227v1, ASM4729169v1, ASM4729180v1, ASM4729225v1, ASM4729195v1, ASM4729166v1, ASM4676607v1, ASM4676609v1, ASM4729219v1, ASM4729197v1, ASM4774805v1) [43] and Asia (NCBI: ASM71030v1, ASM2756380v1).

Good quality genome assemblies generated using a hybrid approach in this study (with N50 values ranging from 0.2 Mb to 11 Mb) enabled analyses of demographic history, gene prediction, and gene family evolution that are difficult to achieve using short-read-only genome data. However, chromosome-level genome assemblies for Asian hornbills are still lacking due to the absence of ultra-long read data. The previously published *B. bicornis* genome assembly is only a short-read data assembly. As a result, it is highly fragmented with 82,936 contigs and a contig N50 value of 34.1 kb. Whereas the hybrid GH assembly in this study is less fragmented (number of contigs 2092) with a contig N50 value of 6.3 Mb. A short-read assembly is not suitable for gene prediction and gene family evolution analyses. Hence, all four hornbill species hybrid genome assemblies from this study provide high-quality reference genomes and insights into demographic history, gene prediction, and gene evolution compared with other Asian hornbill species. The BUSCO scores for gene predictions using GALBA were high (ranging between 68.1% to 95.9%), indicating a high level of completeness. Additionally, OMArk placed all four genomes in this study under the Neognathae lineage, suggesting that annotated genomes are robust and reliable. We also found that assemblers Canu (used for GH) and HifiAsm (used for WH, RNH, MPH) had comparable QV values and 99.9% correctness, indicating that both methods yielded robust assemblies. In comparison with *B. rhinoceros*, genomes from this study showed lower proportions of repeat elements, indicating lineage-specific loss of repetitive elements, large segmental deletions, and gene loss [50, 51]. The repeat elements are crucial in determining genome stability, changes in gene expression, colour polymorphism, and the evolution of gene regulatory networks [52]. The relative prevalence of transposable elements (retroelements and DNA transposons) in all four hornbill genomes was similar to that of other bird species [51]. In future, with more representation of high-quality genomes from the order Bucerotiformes would be essential to study the evolution of repeat elements in depth.

We acknowledge the limitations of our gene orthology assignment and gene family evolution analyses, which are based on a relatively small number of closely related hornbill species. Therefore, these analyses should be considered a preliminary attempt to understand patterns of gene evolution in Asian hornbills. Nevertheless, we identified significant expansion in gene families associated with structural keratin development in Asian hornbills compared to their ancestors. These include genes potentially related to the development and morphology of feathers, beaks, and casques, traits that are highly diverse and characteristic of hornbills. At present, the lack of high-quality annotated genomes for most hornbill species remains a major limitation for more comprehensive analyses of orthology, gene family evolution, and positive selection.

The newly assembled circular mitogenomes are similar in size to those previously reported [14, 15]. The gene lengths and mitogenome organisations of GH and WH were highly comparable to those of the published mitogenomes [14, 15]. The mitogenome assembly sizes in hornbills from this study are comparable to the average for birds (mean = 15,464; range: 13,000–17,000; *n* = 363 bird species) and for Bucerotiformes (13,487–16,045) [41, 42]. When compared across hornbill species, the gene arrangements are highly conserved. However, the gene lengths showed slight variation in a few genes. This intra-species and inter-species mitogenome comparison provides a basis for insights into gene variation, showing evidence of mitochondrial structure diversity.

We observed notable differences in the demographic histories of the four hornbill species. The peak effective population sizes differed across species over the analysed time span, with the WH having at least three times higher peak effective population sizes than its other Himalayan counterparts, the GH and RNH. A previous study found no phylogenetic signal in effective population sizes [12], suggesting that closely related species may differ in their effective population sizes. Large, continuous populations tend to have high genetic diversity and, hence, high Ne [46]. WH is more abundant than RNH and GH in the Eastern Himalaya [53], inhabiting diverse habitats such as low-elevation moist and wet forests in the foothills. A recent synthesis on demographic trends in birds over the past one million years suggested that migratory birds exhibited a longer-term increase in their effective population sizes than non-migratory species [54]. This could provide another line of support for threefold higher Ne in WH, known for long-distance migration. Whereas, GH and RNH are resident species with their distributions restricted to pockets of wet forests.

The different hornbill species responded idiosyncratically to multiple cooling cycles during the Pleistocene. During the mid-Pleistocene (till 400 kya), the Eastern Himalayan region likely maintained humid tropical conditions [55], providing suitable breeding habitats for the WH and supporting a drastic increase in its effective population size. In contrast, GH in the Eastern Himalaya region showed a decline in population size during this time despite similar ecological preferences, highlighting idiosyncratic species responses to the environment. Glacial periods, particularly during the onset of LIG (~120 kya), were associated with reduced wet tropical forest cover, constraining habitat availability for these forest-dwelling birds. Populations of the MPH and GH in the Western Ghats and RNH in the Eastern Himalaya showed gradual declines, a trend observed globally for multiple bird species [12]. Interestingly, the drastic declines in the Eastern Himalayan GH population (~70 kya) aligns with regional evidence for a cool and dry climate [56, 57], however, an earlier sharp decline in the WH around 400 kya may reflect undocumented vegetation shifts. The RNH and, to a lesser extent, the MPH and GH from the Eastern Himalaya exhibited multiple demographic fluctuations, some pre-dating the LIG, pointing to complex interactions between climate, habitat and species-specific histories. While PSMC is applied to infer long-term demographic histories from single diploid genomes, such analyses may not capture within-species variation. Due to logistical constraints, additional high-coverage whole genomes were not available for MPH in the present study.

Population declines during the climatic oscillations of the Pleistocene may indicate that hornbill species are sensitive to human-mediated climate changes, which could potentially alter their habitat availability. Historical climatic shifts and resulting habitat contractions may also have contributed to the fragmented distributions observed in Asian hornbills, with the Great Hornbill representing a notable example. In addition, sea-level fluctuations during the Pleistocene may have isolated island populations across Southeast Asia [10], potentially facilitating diversification among hornbill species in the region [9]. The Late Pleistocene vertebrate assemblages confirm the occurrence of birds, including raptors and hornbills, in Southeast Asia from ~126 kya to 11.7 kya [58, 59]. Hornbill remains recovered from the Niah Cave excavations, dating to ~ 45 kya [60], suggest that humans and hornbills co-occurred in the region during this period. Given traits such as large body size, communal roosting, and incarcerated nesting behaviour, hornbills have been particularly vulnerable to hunting pressures [61, 62]. Although even low levels of hunting can potentially result in local extirpations in contemporary hornbill populations, the extent to which prehistoric human interactions contributed to Late Pleistocene demographic changes remains unclear and warrants further investigation.

## Conclusions

The reference genomes of the *B. bicornis* (GH), *R. undulatus* (WH), *A. coronatus* (MPH), and *A. nipalensis* (RNH) will serve as a resource to further understand the genetic makeup of hornbills. Using these reference genomes, we could reconstruct the demographic histories of these threatened species and understand their population trajectories in response to drivers such as climatic fluctuations and human footprint. It now enables us to investigate comparative genomics and genomic variation across geographic regions and demographic timescales. With the increasing application of genomics into conservation biology, we hope that these reference genomes will act as a tool for conservation prioritization exercises and management for hornbills in the near future.

## Electronic supplementary material

Below is the link to the electronic supplementary material.

Supplementary material 1

## Data Availability

The sample details have been submitted to the National Centre for Biotechnology Information (NCBI) database under the Bioproject PRJNA1345020. The genome assemblies and raw sequence data have been submitted under Accession numbers SRR35817023: SRR35817028, SRR39146887.

## References

[1] Poonswad P, Kemp A, Strange M. Hornbills of the world. A photographic Guide. Draco Publishing and Distribution Pte Limited; 2013.

[2] Kinnaird MF, O’Brien TG. The Ecology and conservation of Asian hornbills: farmers of the forest. University of Chicago Press; 2007.

[3] Naniwadekar R, Shukla U, Isvaran K, Datta A. Reduced hornbill abundance associated with low seed arrival and altered recruitment in a hunted and logged tropical forest. PLoS ONE. 2015;10(3):e0120062. 10.1371/journal.pone.0120062. 10.1371/journal.pone.0120062 PMC4363152 25781944

[4] Shepherd CR, Shepherd L, Rathore D, Mendiratta U. Recent seizures of hornbills trafficked to India. Hornbill Nat Hist Conserv. 2023;4:20–24.

[5] Datta A. Hornbill abundance in unlogged forest, selectively logged forest and a forest plantation in Arunachal Pradesh, India. Oryx. 1998;32(4):285–94. 10.1046/j.1365-3008.1998.d01-58.x.

[6] Sitompul AF, Kinnaird MF, O’Brien TG. Size matters: the effects of forest fragmentation and resource availability on the endemic Sumba hornbill Aceros everetti. Bird Conserv Int. 2004;14(S1):S23–37. 10.1017/S0959270905000201.

[7] Pawar PY, Mudappa D, Raman TRS. Hornbill abundance and breeding incidence in relation to habitat modification and fig fruit availability. Ibis (Lond 1859). 2021;163(2):473–85. 10.1111/ibi.12895.

[8] Dharapuram B, Pawar P, Joshi J, Naniwadekar R. Low mitochondrial genetic diversity in the Narcondam hornbill (*Rhyticeros narcondami*), an island endemic frugivore of ecological importance. Report Submitted To Br Ornithologists’ Union. 2023.

[9] Gonzalez JCT, Sheldon BC, Collar NJ, Tobias JA. A comprehensive molecular phylogeny for the hornbills (Aves: Bucerotidae). Mol Phylogenet Evol. 2013;67(2):468–83. 10.1016/j.ympev.2013.02.012. 23438388 10.1016/j.ympev.2013.02.012

[10] Sammler S, Ketmaier V, Havenstein K, Krause U, Curio E, Tiedemann R. Mitochondrial control region I and microsatellite analyses of endangered Philippine hornbill species (Aves; Bucerotidae) detect gene flow between island populations and genetic diversity loss. BMC Evol Biol. 2012;12(1):203. 10.1186/1471-2148-12-203. 23057730 10.1186/1471-2148-12-203 PMC3532089

[11] Jansamut P, Gale GA, Sukmak M, Wajjwalku W, Punkong C, Kaolim N, et al. Mitogenome-based genetic management of captive Great hornbill in Thailand: implications for reintroduction. Global Ecol Conserv. 2024;51:e02932. 10.1016/j.gecco.2024.e02932.

[12] Nadachowska-Brzyska K, Li C, Smeds L, Zhang G, Ellegren H. Temporal dynamics of avian populations during Pleistocene revealed by whole-genome sequences. Curr Biol. 2015;25(10):1375–80. 10.1016/j.cub.2015.03.047. 25891404 10.1016/j.cub.2015.03.047 PMC4446789

[13] Sammler S, Bleidorn C, Tiedemann R. Full mitochondrial genome sequences of two endemic Philippine hornbill species (Aves: Bucerotidae) provide evidence for pervasive mitochondrial DNA recombination. BMC Genomics. 2011;12(1):1–10. 10.1186/1471-2164-12-35. 10.1186/1471-2164-12-35 PMC3025957 21235758

[14] Chen Y, Li C, Yan H, Xiao H, Chen S. The complete mitochondrial genome sequence of Buceros bicornis (bucerotiformes: bucerotidae). Conserv Genet Resour. 2018;10(3):287–90. 10.1007/s12686-017-0803-4.

[15] Chen Y, Yan H, Sun J, Li C, Xiao H, Chen S. Characterization and phylogenetic analysis of the complete mitochondrial genome sequence of Rhyticeros undulatus (bucerotiformes: bucerotidae). Conserv Genet Resour. 2019;11(1):27–30. 10.1007/s12686-017-0957-0.

[16] Morley RJ. Assembly and division of the South and South-east Asian flora in relation to tectonics and climate change. J Trop Ecol. 2018;34(4):209–34. 10.1017/S0266467418000202.

[17] Hamilton R, Amano N, Bradshaw CJA, Saltré F, Patalano R, Penny D, et al. Forest mosaics, not savanna corridors, dominated in Southeast Asia during the last glacial maximum. Proc Natl Acad Sci USA. 2024;121(1):e2311280120. 10.1073/pnas.2311280120. 10.1073/pnas.2311280120 PMC10769823 38147645

[18] Patel J, Botes A, Mollett J, De Maayer P. Whole genome sequencing, assembly and annotation of the Southern Ground hornbill – Bucorvus leadbeateri. Sci DataSci Data. 2025;12(1):58. 10.1038/s41597-025-04412-2. 10.1038/s41597-025-04412-2 PMC11724890 39799121

[19] Chen S. Ultrafast one-pass FASTQ data preprocessing, quality control, and deduplication using fastp. Imeta. 2023;2(2):e107. 10.1002/imt2.107. 10.1002/imt2.107 PMC10989850 38868435

[20] Koren S, Walenz BP, Berlin K, Miller JR, Bergman NH, Phillippy AM. Canu: scalable and accurate long-read assembly via adaptive k-mer weighting and repeat separation. Genome Res. 2017;27(5):722–36. 10.1101/gr.215087.116. 28298431 10.1101/gr.215087.116 PMC5411767

[21] Cheng H, Jarvis ED, Fedrigo O, Koepfli KP, Urban L, Gemmell NJ, et al. Haplotype-resolved assembly of diploid genomes without parental data. Nat Biotechnol. 2022;40(9):1332–35. 10.1038/s41587-022-01261-x. 35332338 10.1038/s41587-022-01261-x PMC9464699

[22] Zimin AV, Salzberg SL. The genome polishing tool POLCA makes fast and accurate corrections in genome assemblies. PLoS Comput Biol. 2020;16(6):e1007981. 10.1371/journal.pcbi.1007981. 10.1371/journal.pcbi.1007981 PMC7347232 32589667

[23] Manni M, Berkeley MR, Seppey M, Simão FA, Zdobnov EM. BUSCO Update: novel and Streamlined Workflows along with broader and deeper phylogenetic coverage for scoring of Eukaryotic, Prokaryotic, and Viral genomes. Mol Biol Evol. 2021;38(10):4647–54. 10.1093/molbev/msab199. 34320186 10.1093/molbev/msab199 PMC8476166

[24] Challis R, Richards E, Rajan J, Cochrane G, Blaxter BM. BlobToolKit – interactive quality assessment of genome assemblies. G3 Genes|Genomes|Genet. 2020;10(4):1361–74. 10.1534/g3.119.400908. 32071071 10.1534/g3.119.400908 PMC7144090

[25] Rhie A, Walenz BP, Koren S, Phillippy AM. Merqury: reference-free quality, completeness, and phasing assessment for genome assemblies. Genome Biol. 2020;21(1):245. 10.1186/s13059-020-02134-9. 32928274 10.1186/s13059-020-02134-9 PMC7488777

[26] Smit A, Hubley R, Green P. RepeatMasker 4.0. Seattle, WA: Institute for Systems Biology; 2013.

[27] Storer J, Hubley R, Rosen J, Wheeler TJ, Smit AF. The Dfam community resource of transposable element families, sequence models, and genome annotations. Mob DNA. 2021;12(1):2. 10.1186/s13100-020-00230-y. 33436076 10.1186/s13100-020-00230-y PMC7805219

[28] Brůna T, Li H, Guhlin J, Honsel D, Herbold S, Stanke M, et al. Galba: genome annotation with miniprot and AUGUSTUS. BMC Bioinf. 2023;24(1):327. 10.1186/s12859-023-05449-z. 10.1186/s12859-023-05449-z PMC10472564 37653395

[29] Rossier V, Warwick Vesztrocy A, Robinson-Rechavi M, Dessimoz C. Omamer: tree-driven and alignment-free protein assignment to subfamilies outperforms closest sequence approaches. Bioinformatics. 2021;37(18):2866–73. 10.1093/bioinformatics/btab219. 33787851 10.1093/bioinformatics/btab219 PMC8479680

[30] Emms DM, Kelly S. OrthoFinder: phylogenetic orthology inference for comparative genomics. Genome Biol. 2019;20(1):238. 10.1186/s13059-019-1832-y. 31727128 10.1186/s13059-019-1832-y PMC6857279

[31] Sun J, Lu F, Luo Y, Bie L, Xu L, Wang Y. OrthoVenn3: an integrated platform for exploring and visualizing orthologous data across genomes. Nucleic Acids Res. 2023;51(W1):W397–403. 10.1093/nar/gkad313. 10.1093/nar/gkad313 PMC10320085 37114999

[32] Mendes FK, Vanderpool D, Fulton B, Hahn MW. CAFE 5 models variation in evolutionary rates among gene families. Bioinformatics. 2021;36(22–23):5516–18. 10.1093/bioinformatics/btaa1022. 33325502 10.1093/bioinformatics/btaa1022

[33] Kumar S, Suleski M, Craig JM, Kasprowicz AE, Sanderford M, Li M, et al. TimeTree 5: an expanded resource for species divergence times. Mol Biol Evol. 2022;39(8):msac174. 10.1093/molbev/msac174. 10.1093/molbev/msac174 PMC9400175 35932227

[34] Cantalapiedra CP, Hernández-Plaza A, Letunic I, Bork P, Huerta-Cepas J. eggNOG-mapper v2: functional annotation, orthology assignments, and domain prediction at the metagenomic scale. Mol Biol Evol. 2021;38(12):5825–29. 10.1093/molbev/msab293. 34597405 10.1093/molbev/msab293 PMC8662613

[35] Marçais G, Delcher AL, Phillippy AM, Coston R, Salzberg SL, Zimin A. MUMmer4: a fast and versatile genome alignment system. PLoS Comput Biol. 2018;14(1):e1005944. 10.1371/journal.pcbi.1005944. 10.1371/journal.pcbi.1005944 PMC5802927 29373581

[36] Nattestad M, Schatz MC. Assemblytics: a web analytics tool for the detection of variants from an assembly. Bioinformatics. 2016;32(19):3021–23. 10.1093/bioinformatics/btw369. 27318204 10.1093/bioinformatics/btw369 PMC6191160

[37] Danecek P, Bonfield JK, Liddle J, Marshall J, Ohan V, Pollard MO, et al. Twelve years of SAMtools and BCFtools. GigaScience. 2021;10(2):giab008. 10.1093/gigascience/giab008. 10.1093/gigascience/giab008 PMC7931819 33590861

[38] Danecek P, Auton A, Abecasis G, Albers CA, Banks E, DePristo MA, et al. The variant call format and VCFtools. Bioinformatics. 2011;27(15):2156–58. 10.1093/bioinformatics/btr330. 21653522 10.1093/bioinformatics/btr330 PMC3137218

[39] Jin JJ, Yu WB, Yang JB, Song Y, DePamphilis CW, Yi T-S, et al. GetOrganelle: a fast and versatile toolkit for accurate de novo assembly of organelle genomes. Genome Biol. 2020;21(1):241. 10.1186/s13059-020-02154-5. 32912315 10.1186/s13059-020-02154-5 PMC7488116

[40] Bernt M, Donath A, Jühling F, Externbrink F, Florentz C, Fritzsch G, et al. MITOS: improved de novo metazoan mitochondrial genome annotation. Mol Phylogenet Evol. 2013;69(2):313–19. 10.1016/j.ympev.2012.08.023. 22982435 10.1016/j.ympev.2012.08.023

[41] Meng G, Li Y, Yang C, Liu S. MitoZ: a toolkit for animal mitochondrial genome assembly, annotation and visualization. Nucleic Acids Res. 2019;47(11):e63. 10.1093/nar/gkz173. 10.1093/nar/gkz173 PMC6582343 30864657

[42] Muhala V, Guimarães-Costa A, Bessa-Silva AR, Rabelo LP, Carneiro J, Macate IE, et al. Comparative mitochondrial genome brings insights to slight variation in gene proportion and large intergenic spacer and phylogenetic relationship of mudskipper species. Sci Rep. 2024;14(1):3358. 10.1038/s41598-024-52979-4. 38336845 10.1038/s41598-024-52979-4 PMC10858209

[43] Li H, Durbin R. Inference of human population history from individual whole-genome sequences. Nature. 2011;475(7357):493–96. 10.1038/nature10231. 21753753 10.1038/nature10231 PMC3154645

[44] García-Alcalde F, Okonechnikov K, Carbonell J, Cruz LM, Götz S, Tarazona S, et al. Qualimap: evaluating next-generation sequencing alignment data. Bioinformatics. 2012;28(20):2678–79. 10.1093/bioinformatics/bts503. 22914218 10.1093/bioinformatics/bts503

[45] Natesh M, Vinay KL, Ghosh S, Jayapal R, Mukherjee S, Vijay N, et al. Contrasting trends of population size change for two eurasian owlet species—Athene brama and glaucidium radiatum from South Asia over the Late Quaternary. Front Ecol Evol. 2020;8:608339. 10.3389/fevo.2020.608339.

[46] Brüniche-Olsen A, Kellner KF, Belant JL, DeWoody JA. Life-history traits and habitat availability shape genomic diversity in birds: implications for conservation. Proceedings of the Royal Society B: Biological Sciences. 2021: 1961, 288. 10.1098/rspb.2021.1441 PMC8548786 34702080

[47] Bird JP, Martin R, Akçakaya HR, Gilroy J, Burfield IJ, Garnett ST, et al. Generation lengths of the world’s birds and their implications for extinction risk. Conserv Biol. 2020;34(5):1252–61. 10.1111/cobi.13486. 32058610 10.1111/cobi.13486

[48] Feng S, Stiller J, Deng Y, Armstrong J, Fang Q, Reeve AH, et al. Dense sampling of bird diversity increases power of comparative genomics. Nature. 2020;587(7833):252–57. 10.1038/s41586-020-2873-9. 33177665 10.1038/s41586-020-2873-9 PMC7759463

[49] Kapusta A, Suh A, Feschotte C. Dynamics of genome size evolution in birds and mammals. Proc Natl Acad Sci USA. 2017;114(8):E1460–69. 10.1073/pnas.1616702114. 10.1073/pnas.1616702114 PMC5338432 28179571

[50] Zhang G, Li C, Li Q, Li B, Larkin DM, Lee C, et al. Comparative genomics reveals insights into avian genome evolution and adaptation. Science. 2014, Dec, 12;346(6215):1311–20. 10.1126/science.1251385. 25504712 10.1126/science.1251385 PMC4390078

[51] Kapusta A, Suh A. Evolution of bird genomes—a transposon’s-eye view. Ann New Y Acad Sci. 2017, Feb;1389(1):164–85. 10.1111/nyas.13295. 10.1111/nyas.13295 27997700

[52] Schrader L, Schmitz J. The impact of transposable elements in adaptive evolution. Mol Ecol. 2019, Mar;28(6):1537–49. 10.1111/mec.14794. 30003608 10.1111/mec.14794

[53] Naniwadekar R, Datta A. Spatial and temporal variation in hornbill densities in Namdapha Tiger Reserve, Arunachal Pradesh, north-east India. Trop Conserv Sci. 2013, Dec;6(6):734–48. 10.1177/194008291300600603.

[54] Germain RR, Feng S, Buffan L, Carmona CP, Chen G, Graves GR, et al. Changes in the functional diversity of modern bird species over the last million years. Proceedings of the National Academy of Sciences. 2023: (7): e2201945119, 120. 10.1073/pnas.2201945119 PMC9963860 36745783

[55] Khan MA, Mahato S, Spicer RA, Spicer TEV, Ali A, Hazra T, et al. Siwalik plant megafossil diversity in the Eastern Himalayas: a review. Plant Divers. 2023;45(3):243–64. 10.1016/j.pld.2022.12.003. 37397603 10.1016/j.pld.2022.12.003 PMC10311196

[56] Bhattacharyya A, Mehrotra N, Shah SK, Basavaiah N, Chaudhary V, Singh IB. Analysis of vegetation and climate change during Late Pleistocene from Ziro Valley, Arunachal Pradesh, Eastern Himalaya region. Quaternary Sci Rev. 2014;101:111–23. 10.1016/j.quascirev.2014.07.008.

[57] Kar R, Quamar MF. Late Pleistocene–Holocene vegetation and climate change from the Western and Eastern Himalaya (india): palynological perspective. Curr Sci. 2020;119(2):195–218. 10.18520/cs/v119/i2/195-218.

[58] Meijer HJM. The avian fossil record in insular Southeast Asia and its implications for avian biogeography and palaeoecology. PeerJ. 2014;2:e295. 10.7717/peerj.295. 10.7717/peerj.295 PMC3961167 24688871

[59] Barton H, Piper PJ, Rabett R, Reeds I. Composite hunting technologies from the terminal Pleistocene and early Holocene, Niah Cave, Borneo. J Archaeolog Sci. 2009;36(8):1708–14. 10.1016/j.jas.2009.03.027.

[60] Piper PJ, Rabett RJ, Kurui EB. Late pleistocene and early holocene forager organizations in Southeast Asia using community, composition and structural variation in terminal Pleistocene vertebrate assemblages to identify human hunting behaviour at the Niah caves, Borneo. BIPPA. 2008;2888–98. 10.7152/bippa.v28i0.12021.

[61] Tyrberg T. The Late Pleistocene continental avian extinction - an evaluation of the fossil evidence. Oryctos. 2008;7:249–69.

[62] Dasgupta S, Hilaluddin. Differential effects of hunting on populations of hornbills and imperial pigeons in the rainforests of the Eastern Indian Himalaya. Indian For. 2012;138:902–09.

